# Clinical and genetic characteristics of and diagnostic insights on KBG syndrome in Chinese patients: a retrospective study and literature review

**DOI:** 10.1186/s13023-026-04305-6

**Published:** 2026-03-26

**Authors:** Shi-Qi Wang, Sheng-Nan Wu, Hai-Yan Wei, Nan Lv, Li-Fang Song, Dong-Xiao Li, Chang Su, Yong-Xing Chen

**Affiliations:** 1 Department of Endocrinology, Genetics and Metabolism, Children’s Hospital Affiliated to Zhengzhou University, Henan Children’s Hospital, Zhengzhou Children’s Hospital, Zhengzhou, Henan China; 2 Tianjian Laboratory of Advanced Biomedical Sciences, Academy of Medical Sciences, Zhengzhou University, Zhengzhou, Henan China; 3 Zhengzhou Children’s Hospital Rehabilitation Center, Children’s Hospital Affiliated to Zhengzhou University, Henan Children’s Hospital, Zhengzhou Children’s Hospital, Zhengzhou, Henan China; 4 Department of Neurology, Children’s Hospital Affiliated to Zhengzhou University, Henan Children’s Hospital, Zhengzhou Children’s Hospital, Zhengzhou, Henan China; 5 Henan Children’s Neurodevelopment Engineering Research Center, Children’s Hospital Affiliated to Zhengzhou University, Henan Children’s Hospital, Zhengzhou Children’s Hospital, Zhengzhou, Henan China; 6 Department of Endocrinology, Genetics and Metabolism, Beijing Children’s Hospital, Capital Medical University, National Center for Children’s Health, Beijing, China

**Keywords:** KBG syndrome, ANKRD11 variation, 16q24.3 deletion, Phenotypic difference, Perinatal

## Abstract

**Background:**

Most published cohorts of individuals with KBG are Caucasian which limits the understanding of the genetic diversity and clinical presentation across ethnicities. Herein, we report Chinese patients with KBGS, summarise their clinical and genetic characteristics, and compare their characteristics with those of non-Chinese patients. We retrospectively analysed the data of 65 Chinese patients, identified 33 previously unreported cases, and compared their data with those of 519 non-Chinese patients.

**Results:**

We noted the most prevalent features in Chinese patients. Large and/or prominent ears, triangular faces, anteverted nares, synophrys, long philtrum, low hairline, spinal anomalies, and recurrent otitis media differed significantly among ethnicities. Among the 51 patients with the c.1903_1907del variant, there were differences across ethnicities; the c.1903_1907del and non-c.1903_1907del variants differed among Chinese patients. Rare phenotypes were identified in Chinese patients. The incidence of IUGR, polyhydramnios, abnormal renal and brain morphology, abnormal cardiovascular system morphology, preterm birth, and higher NT was higher in all patients. Genetic sequencing revealed 14 unreported variants. Eight patients were treated with recombinant human growth hormone and had increased height without significant adverse effects.

**Conclusions:**

Our findings support the consideration of KBGS in the differential diagnosis when specific prenatal features (such as IUGR and polyhydramnios) are present. In such scenarios, referral for genetic counseling to discuss the option of testing for ANKRD11 variants and 16q24.3 microdeletions is a prudent clinical step.Overall, Chinese patients with KBGS appear to tolerate growth hormone treatment, but further studies are needed to confirm its long-term safety and efficacy.

**Supplementary Information:**

The online version contains supplementary material available at https://doi.org/10.1186/s13023-026-04305-6.

## Background

KBG syndrome (KBGS) was named after the initials of three unrelated families first reported by Herrmann, et al. in 1975 and is characterised by neurodevelopmental delay, short stature, characteristic features, macrodontia, and skeletal abnormalities [1]. In 2011, Sirmaci et al. [2] confirmed that KBGS is caused by haploinsufficiency of ankyrin repeat domain 11 (ANKRD11) variation or 16q24.3 deletion containing *ANKRD11*, which is inherited in an autosomal dominant form. ANKRD11 is a transcriptional co-regulator of histone acetylation, which affects the nervous system development [3]. P21 is involved in early chondrogenic differentiation and ANKRD11 can enhance the retrotranscriptional activation of *P21* and participate in chondrogenic differentiation [4]. The ANKRD11 variant may cause impaired neurological and bone development, resulting in various symptoms.

The incidence rate of KBGS, a rare genetic disorder, remains unknown, and over 700 cases have been reported worldwide (Human Genetic Variant Database HGMD professional version, accessed in March 2025). However, owing to phenotype heterogeneity and the possible somatic mosaicism of *ANKRD11*, the penetrance of KBGS may differ, possibly resulting in its underestimation [5].

There are no unified diagnostic criteria for KBGS. The newly recommended clinical diagnostic criteria was that patients should meet two or more major criteria or one major standard plus two or more minor criteria. The major criteria are (1) macrodontia of the upper central incisors, (2) developmental delay related to behavioural challenges, mild/moderate intellectual disability, or learning difficulties, (3) craniofacial anomalies (e.g. triangular face, synophrys, prominent nasal bridge, anteverted nares, long philtrum, and thin vermilion of the upper lip), (4) postnatal short stature, and (5) first-degree relatives with KBGS. The minor criteria are (1) conductive hearing loss caused by recurrent otitis media, (2) palatal abnormalities, (3) hair abnormalities (e.g. low hairline, coarse hair), (4) delayed bone age (below average > 2 SD), (5) skeletal anomalies, namely large anterior fontanel with delayed closure, hand abnormalities, thoracolumbar deformities, and scoliosis, (6) abnormal electroencephalogram (EEG) findings with or without epilepsy, (7) feeding difficulties, and (8) male cryptorchidism [6].

Previous studies did not find any differences between Chinese and non-Chinese patients [7, 8]. Some researchers have attempted to establish genotypic and phenotypic correlations related to ANKRD11 variant loci or types, such as the effects of c.1903_1907del variant on hearing and nervous system; however, their conclusions were inconsistent, and there was no significant difference [9, 10]. possibly owing to the limited cohort size. In addition, studies on genotypic–phenotypic associations in Chinese patients are lacking.

Therefore, in this study, we aimed to report Chinese patients with KBGS, summarise their clinical and genetic characteristics, and compare their characteristics with those of non-Chinese patients. The study included 65 Chinese KBGS patients, comprising 33 novel cases and 32 previously published cases. This is the largest cohort of Chinese patients analysed to date, aiming to delineate the clinical and genetic spectrum of KBGS in Chinese patients, compare phenotypic characteristics with other ethnic populations, and explore potential genotype-phenotype correlations, thereby providing deeper insights for diagnosis.

## Methods

### Patients

Sixty-five Chinese patients with *ANKRD11* variation or 16q24.3 deletion containing *ANKRD11* were retrospectively analysed, and questionnaires (Additional File 1) were formulated according to the diagnostic criteria for KBGS in 2017 [6], clinical phenotypes mentioned in previous literature, and other clinical phenotypes of patients encountered in our study. These patients were recruited through a retrospective analysis of the medical records of patients with KBGS, using different modes such as face-to-face diagnosis and video calling. The participants were from various regions in China, such as Henan, Beijing, Hubei, Anhui, Shandong, Zhejiang, Jiangxi, and Chongqing. For 33 unreported cases, informed consent of each patient’s legal guardian was obtained, and each patient was evaluated by a professional endocrinologist and geneticist, with their clinical data being meticulously collected. The study protocol and related procedures were approved by ethics committee of Henan Children’s Hospital, Zhengzhou, Henan, China (No. 2024-K-099). Informed consent was obtained from all patients and their parents before they participated in the study. The research process was carried out in strict accordance with the tenets of the Declaration of Helsinki. This study was conducted between January 2020 and December 2024. Patient enrolment, treatment administration, and follow-up assessments were carried out during this period.

### Literature review

With the keywords ‘KBG’ OR ‘ANKRD11’ OR ‘16q24.3 deletion’, literature reports on KBGS between 2010 and December 2024 were searched in PubMed, Ovid Medicine, and Springer. According to the questionnaire and the comprehensiveness of the information that was developed here, 519 non-Chinese patients were screened and their clinical phenotypes and genotypes were collected and compared with those of Chinese patients.

Venous anticoagulation blood was collected from the 33 unreported patients for whole exome sequencing (WES), and Sanger sequencing was performed to verify the variation in candidate genes. In our new cohort, in case 2 and case 33, parental testing could not be conducted to determine the origin of the variation. The pathogenicity of the variant sites was evaluated following the standards and guidelines for the interpretation of sequence variants by a joint consensus recommendation of the American College of Medical Genetics and Genomics (ACMG)and the Association for Molecular Pathology. All variants were referred to the genomic constructs hg19 and ANKRD11 transcript NM_013275.6.

### Assessment of neurodevelopmental status

The evaluation of intellectual disability (ID) and developmental delay was based on a combination of standardized psychological assessments and clinical evaluation. For younger children, the Griffiths Mental Development Scales were used to obtain a developmental quotient (DQ). For older children, the Wechsler Intelligence Scales (Wechsler Preschool and Primary Scale of Intelligence or Wechsler Intelligence Scale for Children) were administered to obtain a full-scale intelligence quotient (IQ). For patients who did not undergo formal standardized testing, the level of developmental delay or intellectual disability was categorized by experienced pediatric neurologists or developmental pediatricians based on comprehensive clinical assessments of adaptive functioning, academic achievement, and clinical observation. It should be noted that not all patients in this retrospective cohort, particularly those from the literature review, had undergone comprehensive neuropsychological testing, which is a limitation of this study.

### Intervention and follow-up

Eight patients diagnosed with KBGS were treated with recombinant human growth hormone (rhGH). Among them, the height of 5 patients was less than − 2SD, meeting the criteria for short stature. The remaining three patients, although they did not reach this specific height threshold, after extensive discussions and at the strong request of their parents, began to receive treatment aimed at improving their children’s height and cognition. Before commencing, obtain written informed consent forms from the guardians of all participants, which include detailed discussions on the main goal of the treatment being to increase height, its potential risks, and the uncertainty of its treatment for KBGS improving cognition. Recombinant human growth hormone (rhGH) is administered subcutaneously daily at 0.075 to 0.100 IU/kg.The dose was adjusted according to the individual patient’s weight, growth response and insulin-like growth factor 1 (IGF-1) level.

All patients treated with rhGH were followed up every 3 to 6 months. The assessment includes measuring height, weight, growth rate, monitoring liver function, kidney function, thyroid function and blood sugar levels.

### Data analysis

The data were analysed using IBM SPSS Statistics 22 (IBM Corp., Armonk, NY, USA). We used the Chi-square test or Fisher’s exact test to calculate the frequency of phenotypic features between the groups. *P* < 0.05 indicated a significant difference.

### Ethics declaration

The study protocol and related procedures were approved by ethics committee of Henan Children’s Hospital, Zhengzhou, Henan, China (No. 2024-K-099). Informed consent was obtained from all patients and their parents before they participated in the study.

## Results

### General information on the 33 unreported Chinese patients in this cohort

The general information of the patients is presented in Table 1. The 33 unreported Chinese patients were from 31 families, 2 of whom were monozygotic twins. There were 23 male and 10 female patients, with a current age range of 9 months to 12 years and 3 months. Their age at the first visit ranged from 1 month to 8 years. Developmental delays (54.5%, 18 of 33) were the most common complaints for the first visit, followed by seizures (18.1%, 6 of 33) and short stature (15.2%, 5 of 33). The age at diagnosis ranged from 1 month to 8 years and 4 months. Data were collected from 25 patients with a mean independent walking age of 17.6 months.

**Table 1:** General information on the 33 unreported Chinese patients in this cohort

Patient ID	Sex	Age	Reasons for the first visit	Age at first visit	Age at diagnosis	Primary presentation at diagnosis	Antenatal examination anomalies
P1	F	2 years, 4 months	Motor development delay	8 months	1 year	Motor development delay	+(single umbilical artery)
P2	F	1 year, 7 months	Short limbs	1 month	1 month	Short limbs	+(Higher NT non-display of nasal bone short limbs IUGR)
P3	F	1 year, 9 months	Motor development delay	9 months	10 months	Global development delay	+ (Hydramnios non-display of nasal bone Thickened placenta)
P4	M	2 years, 3 months	Motor development delay	1 year, 2 months	1 year, 2 months	Global development delay	+(Hydramnios lateral ventricle broadening high risk for Down’s screening)
P5	M	6 years, 11 months	Seizure	6 years, 1 month	6 years, 1 month	Epilepsy	+(Umbilical cord torsion)
P6	F	3 years, 2 months	Global development delay Large head circumference	7 months	10 months	Global development delay	+(Atrial septal defect)
P7	F	1 year, 2 months	Global development delay	2 months	5 months	Global development delay	+(Hydramnios Higher NT PLSVC)
P8	M	4 years, 10 months	Global development delay Large anterior fontanelle with delayed closure	1 year, 2 months	1 year, 7 months	Global development delay	+(IUGR)
P9	M	2 years, 11 months	Feeding difficulty	3 months	11 months	Global development delay	+(IUGR)
P10	M	4 years, 3 months	Global development delay	1 year	3 years, 6 months	Global development delay	+(lateral ventricle broadening)
P11	M	4 years, 3 months	Global development delay	1 year	3 years, 6 months	Global development delay	+(lateral ventricle broadening)
P12	M	3 years, 10 months	Global development delay Fundus lesions	5 months	6 months	Global development delay Fundus lesions	+(cardiac abnormalities)
P13	M	12 years, 3 months	Learning difficulty	5 years	7 years	ADHD	+(single umbilical artery)
P14	F	6 years	Hearing loss Recurrent otitis media	4 years	5 years	Conductive deafness Clinodactyly of the 5th finger Motor development delay	+(short umbilical cord)
P15	M	9 months	Motor development delay	8 months	9 months	Motor development delay	−
P16	M	1 year, 6 months	Seizure	6 months	7 months	Epilepsy	−
P17	M	3 years, 5 months	Motor development delay	7 months	8 months	Motor development delay	−
P18	M	2 years, 3 months	Seizure	5 months	5 months +	Infantile spasms	−
P19	M	2 years, 2 months	Blepharoptosis Global development delay	6 months	1 year	Global development delay Craniofacial anomalies	+ (high risk for Down’s screening)
P20	M	8 years	Seizure	4 years, 6 months	4 years, 6 months	Language delay Epilepsy	−
P21	F	9 years	Seizure	8 years	8 years	Epilepsy Craniofacial anomalies	+(lateral ventricle broadening)
P22	M	3 years, 3 months	Global development delay	7 months	7 months	Global development delay	+(Hydramnios VSD short umbilical cord)
P23	M	2 years, 10 months	Motor development delay Middle ear effusion	6 months	8 months	Motor development delay Head haemangioma Umbilical hernia Hearing loss	+(Hydramnios)
P24	M	1 year, 11 months	Motor development delay Short stature	6 months	11 months	Motor development delay Short stature	+(Hydramnios Higher NT lateral ventricle broadening)
P25	M	5 years, 3 months	Short stature	5 months	6 months	Short stature Craniofacial anomalies	+(Less foetal movement)
P26	M	3 years, 4 months	Low body weight Cryptorchidism VSD	2 months	3 months	Low body weight Cryptorchidism VSD	+(IUGR)
P27	M	3 years, 4 months	Genetic testing	3 months	3 months	Genetic verification diagnosis	−
P28	M	6 years, 3 months	Short stature	2 years, 2 months	4 years, 2 months	Short stature Craniofacial anomalies	−
P29	M	4 years, 11 months	Short stature Global development delay	2 years, 6 months	2 years, 7 months	Short stature Global development delay Short limbs Grumpy	+(IUGR)
P30	M	1 year	Global development delay	7 months, 7 days	8 months, 17 days	Global development delay Craniofacial anomalies Cryptorchidism	−
P31	F	5 years, 4 months	Febrile seizure	3 years, 3 months	3 years, 5 months	Global development delay Epilepsy	+(Higher NT)
P32	F	2 years	Motor development delay	1 year, 4 months	1 year, 5 months	Global development delay	+(hydropericardium)
P33	F	8 years, 4 months	Short stature	6 years, 9 months	8 years, 4 months	Short stature	+(intrauterine hypoxia)

NT, nuchal translucency; IUGR, intrauterine growth retardation; VSD, ventricular septal defect; PLSVC, persistent left superior vena cava; ADHD, attention-deficit hyperactivity disorder

### Comparison of some clinical manifestations in different groups

The clinical characteristics of all the symptomatic patients in the different groups are summarised in Table 2. The most common clinical symptoms in the 65 Chinese patients were delayed bone age (80.7%), large and/or prominent ears (78.9%), macrodontia (69.1%), brachydactyly and/or clinodactyly (68.0%), history of developmental delay (66.0%), abnormal cerebral magnetic resonance imaging (MRI) findings (56.3%), no/borderline intellectual disability (ID) (54.3%), visual challenges (52.9%), short stature (≤ P3) (51.6%), and hypertelorism (51.4%). The primary characteristics of the 519 non-Chinese patients were a history of developmental delay (94.0%), bushy and/or thick eyebrows (79.1%), macrodontia (77.5%), long philtrum (63.8%), brachydactyly and/or clinodactyly (62.2%), anteverted nares (59.3%), large and/or prominent ears (57.4%), triangular face (56.9%), abnormal cerebral MRI findings (56.1%), visual complications (54.9%), mild ID (53.9%), short stature (≤ P3) (53.0%), synophrys (51.4%), and low hairline (51.1%).

**Table 2:** Clinical characteristics of symptomatic patients in different groups

	Non-Chinese			Chinese		
	Total (*N* = 519)	Non-c. 1903_1907del (*n* = 473)	c.1903_1907del (*n* = 46)	Total (*N* = 65)	Non-c. 1903_1907del(*n* = 60)	c.1903_1907del (*n* = 5)
Male/Female	284/226	265/208	19/18	39/26	37/23	2/3
	n(%)	n(%)	n(%)	n(%)	n(%)	n(%)
Idiofacies						
Triangular face	120/211(56.9)	112/197(56.7)	8/14(57.1)	13/38(34.2)^b^	11/36(30.5)	2/2(100)
Synophrys	90/175(51.4)	83/157(52.9)	7/18(38.9)	8/35(22.9)^b^	8/33(24.2)	0/2(0.0)
Bushy and/or thick eyebrows	178/225(79.1)	169/209(80.9)	9/16(56.3)a	15/37(40.5)^b^	15/35(42.9)	0/2(0.0)
Hypertelorism	109/223(48.8)	101/204(45.9)	8/19(42.1)	18/35(51.4)	18/33(54.5)	0/2(0.0)
Large and/or prominent ears	116/202(57.4)	109/191(57.1)	7/11(63.6)	30/38(78.9)^b^	29/36(80.6)	1/2(50.0)
Low-set ears	31/101(30.6)	26/93(28.0)	5/8(62.5)	2/33(6.1)^b^	2/33(6.1)	0/0(0.0)
Prominent nose	46/129(35.6)	41/120(34.2)	5/9(55.6)	9/35(25.7)	9/33(27.3)	0/2(0.0)
Anteverted nares	127/214(59.3)	118/200(59.0)	9/14(64.3)	12/34(35.3)^b^	12/32(37.5)	0/2(0.0)
Long philtrum	153/240(63.8)	140/221(63.3)	13/19(68.4)	14/35(40)^b^	14/33(42.4)	0/2(0.0)
cleft palate	15/216(6.9)	14/193(7.3)	1/23(4.3)	0/35(0.0)	0/31(0.0)	0/4(0.0)
Thin upper lip	97/231(42.0)	86/214(69.4)	11/17(64.7)^a^	16/35(45.7)	16/33(48.5)	0/2(0.0)
Low hairline	89/174(51.1)	81/156(51.9)	8/18(44.4)	5/35(14.3)^b^	5/35(14.3)	0/0(0.0)
Macrodontia	279/360(77.5)	251/324(77.5)	28/36(77.8)	39/61(69.1)^b^	36/57(63.2)	3/4(75.0)
Other dental anomalies	102/239(42.7)	97/207(46.9)	15/32(46.9)	28/58(48.3)	26/54(48.1)	2/4(50.0)
Skeletal anomaly						
Short stature (≤ p3)	196/370(53.0)	181/340(53.2)	15/30(50.0)	32/62(51.6)	32/57(56.1)	0/5(0.0)
Larger anterior fontanels or delayed closure	58/276(21.0)	51/260(19.6)	7/16(43.8)^a^	27/63(42.9)^b^	26/61(42.6)	1/2(50.0)
Vertebral anomaly	30/144(20.8)	26/128(20.3)	4/16(25.0)	9/47(19.1)	7/43(16.3)	2/4(50.0)
Spinal anomaly	59/280(21.1)	57/265(21.5)	2/15(13.3)	6/53(11.3)^b^	6/51(11.8)	0/2(0.0)
Short neck webbed	32/120(26.7)	28/111(25.2)	4/9(44.4)	4/34(11.8)^b^	4/32(12.5)	0/2(0.0)
Rib anomaly	36/146(24.7)	33/132(25.0)	3/14(21.4)	11/46(23.9)	11/44(25.0)	0/2(0.0)
Sternal anomaly	14/80(17.5)	14/71(19.7)	0/9(0.0)	7/47(14.9)	7/47(14.9)	0/0(0.0)
Brachydactyly and/or clinodactyly	250/402(62.2)	226/369(61.2)	24/33(72.7)	34/50(68.0)	32/46(69.6)	2/4(50.0)
Hip anomaly	17/127(13.4)	17/126(13.5)	0/1(0.0)	4/33(12.1)	4/33(12.1)	0/0(0.0)
Delayed bone age	93/220(42.3)	82/198(41.4)	11/22(50.0)	21/26(80.7)^b^	21/24(87.5)	0/2(0.0)^d^
Neurological abnormalities						
ADHD symptoms	107/240(44.7)	99/223(44.4)	8/17(47.1)	16/44(36.4)	14/41(34.1)	2/3(66.7)
ASD symptoms	118/316(37.3)	114/302(37.7)	4/14(28.6)	4/44(9.1)^b^	3/43(7.0)	1/1(100.0)
Anxiety	40/206(19.4)	40/203(19.7)	0/3(0.0)	2/33(6.1)	2/33(6.1)	0/0(0.0)
Shy	7/127(5.5)	6/119(5.0)	1/8(12.5)	8/33(24.2)^b^	8/33(24.2)	0/0(0.0)
Aggressive behaviour	46/241(19.1)	46/238(19.3)	0/3(0.0)	8/34(23.5)	8/34(23.5)	0/0(0.0)
Difficulty in social	22/150(14.7)	21/146(19.3)	1/4(25.0)	6/33(18.2)	6/33(18.2)	0/0(0.0)
ID						
No/Borderline	41/232(17.7)	35/212(16.5)	6/20(30.0)	19/35(54.3)^b^	19/34(55.9)	0/1(0.0)
Mild	125/232(53.9)	117/212(55.2)	8/20(40.0)	13/35(37.1)	12/34(35.3)	1/1(100.0)
Moderate	55/232(23.7)	50/212(23.6)	5/20(25.0)	3/35(8.6)^b^	3/34(8.8)	0/1(0.0)
Severe	11/232(4.7)	10/212(4.7)	1/20(5.0)	0/35(0.0)	0/34(0.0)	0/1(0.0)
Seizures	121/379(31.9)	112/347(32.3)	9/32(28.1)	10/56(17.9)^b^	7/51(13.7)	3/5(60.0)^d^
Abnormal EEG	20/72(27.8)	17/57(29.8)	3/15(20.0)	14/39(35.9)	11/35(31.4)	3/4(75.0)^c^
Abnormal cerebral MRI	120/214(56.1)	107/192(55.7)	13/22(59.1)	18/32(56.3)	17/28(60.7)	1/4(25.0)
History of developmental delay	233/248(94.0)	209/214(97.7)	24/34(70.6)^a^	35/53(66.0)^b^	30/48(62.5)	5/5(100.0)
Congenital heart defects	145/428(33.9)	139/393(35.4)	6/35(17.1)^a^	20/57(35.1)	18/52(34.6)	2/5(40.0)
Recurrent otitis media	44/118(37.3)	40/104(38.5)	4/14(28.6)	7/43(16.3)^b^	7/41(17.1)	0/2(0.0)
Hearing loss	144/325(44.3)	132/293(45.1)	12/32(37.5)	14/44(31.8)	14/39(35.9)	0/5(0.0) ^c^
Visual problems	100/182(54.9)	92/157(58.6)	8/25(32.0) ^a^	27/51(52.9)	25/48(52.1)	2/3(66.7)
Feeding difficulties	112/262(42.7)	105/242(43.4)	7/20(35.0)	18/44(40.9)	17/42(40.5)	1/2(50.0)
Cryptorchidism	66/186(35.5)	61/170(35.9)	5/16(31.2)	6/31(19.4)	6/28(21.4)	0/3(0.0)
Precocious puberty in males	8/66(12.1)	8/66(12.1)	0/0(0.0)	1/30(3.3)	1/30(3.3)	0/0(0.0)
Precocious puberty in females	13/138(9.4)	13/138(9.4)	0/0(0.0)	1/16(6.3)	0/12(0.0)	1/4(25.0)

ASD, autism spectrum disorder; ID, intellectual disability; EEG, Electroencephalogram; MRI, magnetic resonance imaging

Data are presented as n(%). ^a^ In the non-Chinese patient group, the difference between c.1903_1907del and non-c.1903_1907del gene variations was statistically significant. ^b^The difference between Chinese and non-Chinese patients was statistically significant. ^c^In patients with the c.1903_1907del gene variant, the difference between Chinese and non-Chinese patients was statistically significant. ^d^ In the Chinese patient group, the difference between c.1903_1907del and non-c.1903_1907del gene variations was statistically significant

Statistical analysis revealed several significant phenotypic differences (*P* < 0.05) between Chinese and non-Chinese patients. The Chinese cohort exhibited a higher incidence of large and/or prominent ears, larger anterior fontanels or delayed closure, and delayed bone age. Conversely, symptoms of autism spectrum disorder (ASD), moderate intellectual disability (ID), epilepsy, a history of developmental delay, and recurrent otitis media were all less prevalent among Chinese patients.

Among the 51 patients with the symptomatic c.1903_1907del variant, Chinese patients were more likely to have EEG abnormalities (75.0%) and less likely to have hearing loss (0.0%). In all non-Chinese patients, those with c.1903_1907del had a higher incidence of larger anterior fontanels or delayed closure and a lower incidence of thick and/or thick eyebrows, thin upper lips, a history of developmental delay, congenital heart defects, and vision challenges. In Chinese patients, the incidence of epilepsy was significantly higher in patients with c.1903_1907del, whereas that of delayed bone age was low, and the differences were statistically significant.

### Comparison of perinatal problems between the Chinese and non-Chinese patients

**Fig. 1:**
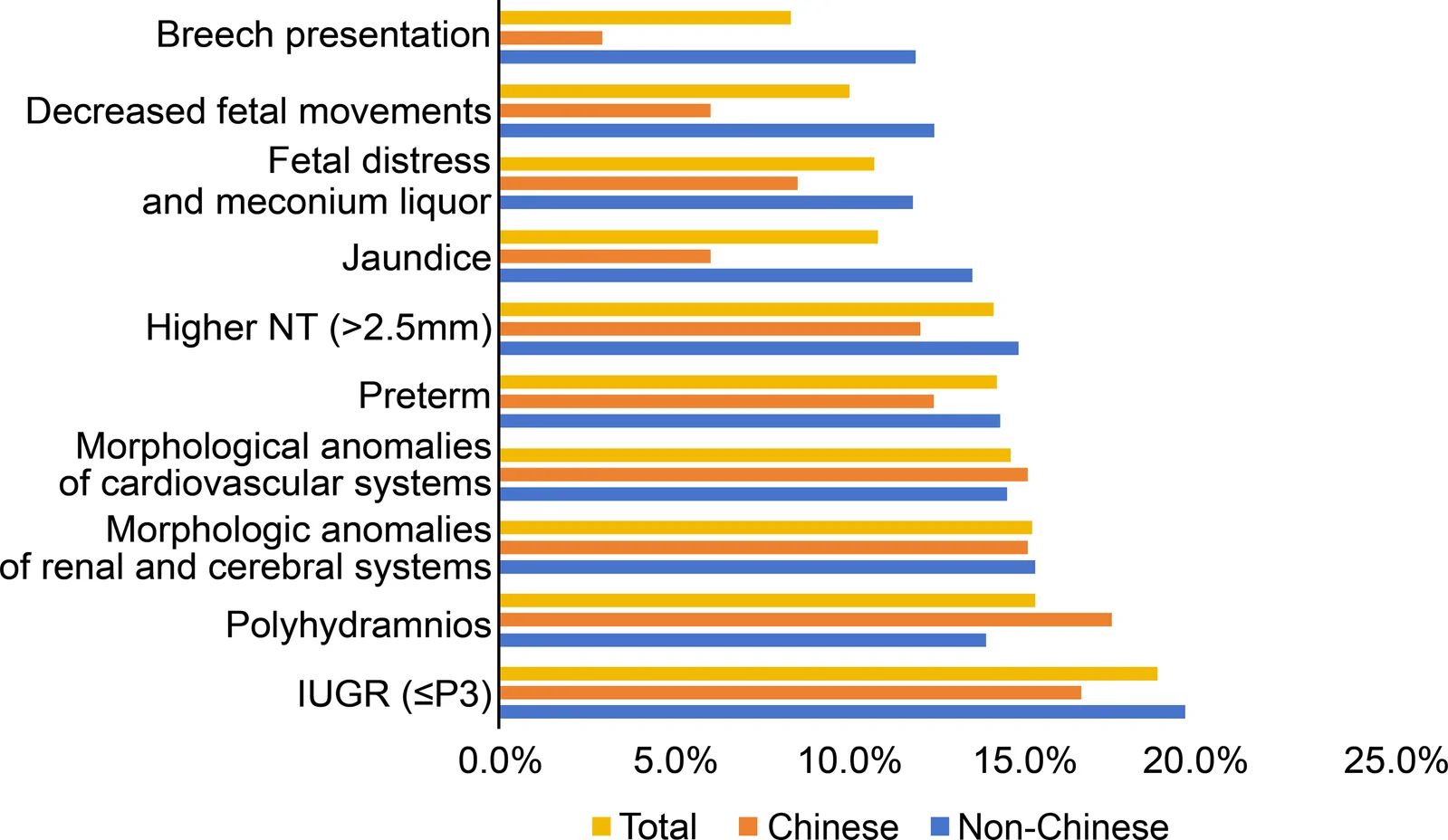
Comparison of perinatal challenges between Chinese and non-Chinese patients

There were no significant differences in perinatal challenges between the groups, and the primary perinatal complications were IUGR, polyhydramnios, morphologic anomalies of the renal, cerebral, or cardiovascular systems, preterm birth, and higher NT (Fig. 1, Additional File 2).

### Molecular findings of the Chinese and non-Chinese patients

The WES showed that 30 of the 33 patients had heterozygous *ANKRD11* variations. Furthermore, 25 variants were identified, 14 of which had not been previously reported. Five pairs of patients who carried the same pathogenic ANKRD11 variant within each pair, two of whom were monozygotic twins. Patient 2 had a microdeletion of 0.092 Mb in 16q24.3, and Patients 19 and 33 had heterozygous deletions in 16q24.2-q24.3. In addition to *ANKRD11* variations, Patient 30 had a 0.22-Mb duplication of the Yq11.223 region, the significance of which was unclear. The variation site was in exon 9 in 23 patients, exon 6 in 2, intron 9 in 2, exon 10 in 1, exon 12 in 1, and exon 13 in 1. Three cases were rated as likely pathogenic, whereas the others were pathogenic. The parents were not verified in Case 2 and the father was not verified in Case 33 for family reasons; the rest had de novo variations.

**Fig. 2:**
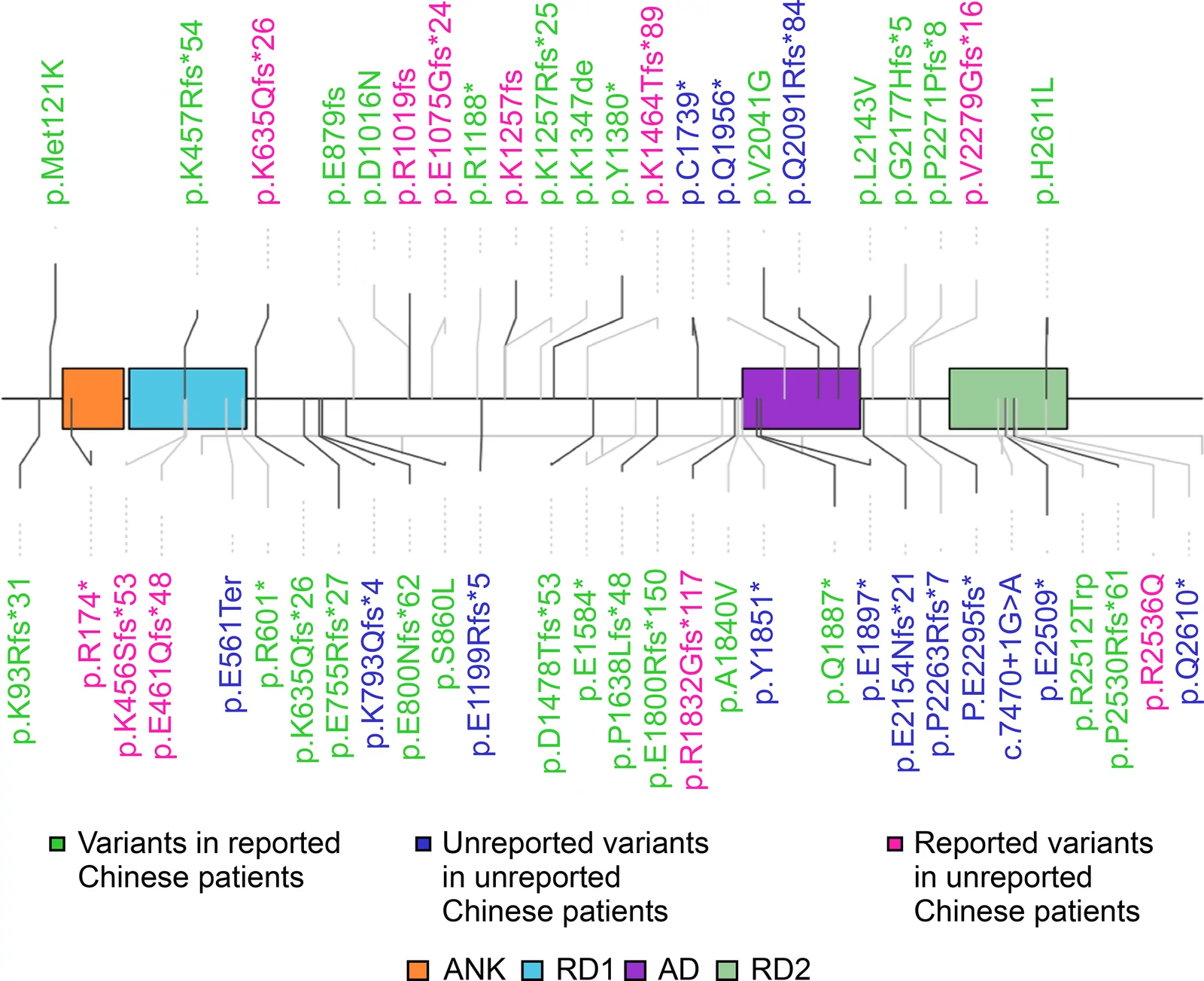
Molecular phenotypes of Chinese patients

Among the 65 Chinese patients, 60 had *ANKRD11* variations, including 33 frameshift, 14 nonsense, 11 missense, and 2 splicing (Fig. 2) (Additional File 3). The other five patients were identified with 16q24.3 deletions that contain the ANKRD11gene.

In all patients, there were 490 ANKRD11 variants, with 289 variant sites. The most common variant was frameshift, followed by nonsense and missense, and a few were splicing. There was no significant difference in the variant rates of ANKRD11 between Chinese and non-Chinese patients (92.3% vs. 83.1%). c.1903_1907del was the most common variant in Chinese and non-Chinese patients, with 51 cases.

### Follow-up and outcome evaluations

Nine of the thirty-three patients had seizures. Four patients presented with tonic seizures, onset age of 2 months to 8 years, and generally, sporadic seizures occurred from 1 month to 6 months after the disease onset. Three of them experienced no recurrence after 6 months of sodium valproate treatment, and one exhibited no recurrence after traditional Chinese medicine treatment. Two patients showed febrile seizures, whose age of onset was approximately 1 year, and the oldest was 5 years and 3 months old, whose febrile seizures had resolved spontaneously. One participant had acoustic reflex myoclonus, which began at the age of 6 months and occurred frequently during the first month of the disease, with dozens of seizures occurring daily, each lasting approximately 1 s. We followed up with the patient for 1 year and found that after 1 week of sodium valproate treatment, there was no recurrence. Patient 18 had infantile spasm (which began at the age of 5 months) and had daily seizures for approximately 1 month, which was controlled after 1 month of medication, and the drug administration was discontinued at the age of 2 years and 3 months. Patient 31 presented with Lennox-Gastaut syndrome (LGS), which started at 3 years and 3 months and lasted 25 months, with daily seizures in various forms, including tonic, clonic, and tonic-clonic. After treatment with levetiracetam, valproate sodium, and lamotrigine, similar seizures persisted daily. Notably, the gene variation in both was c.1903_1907del (p.K635Qfs*26).

**Fig. 3:**
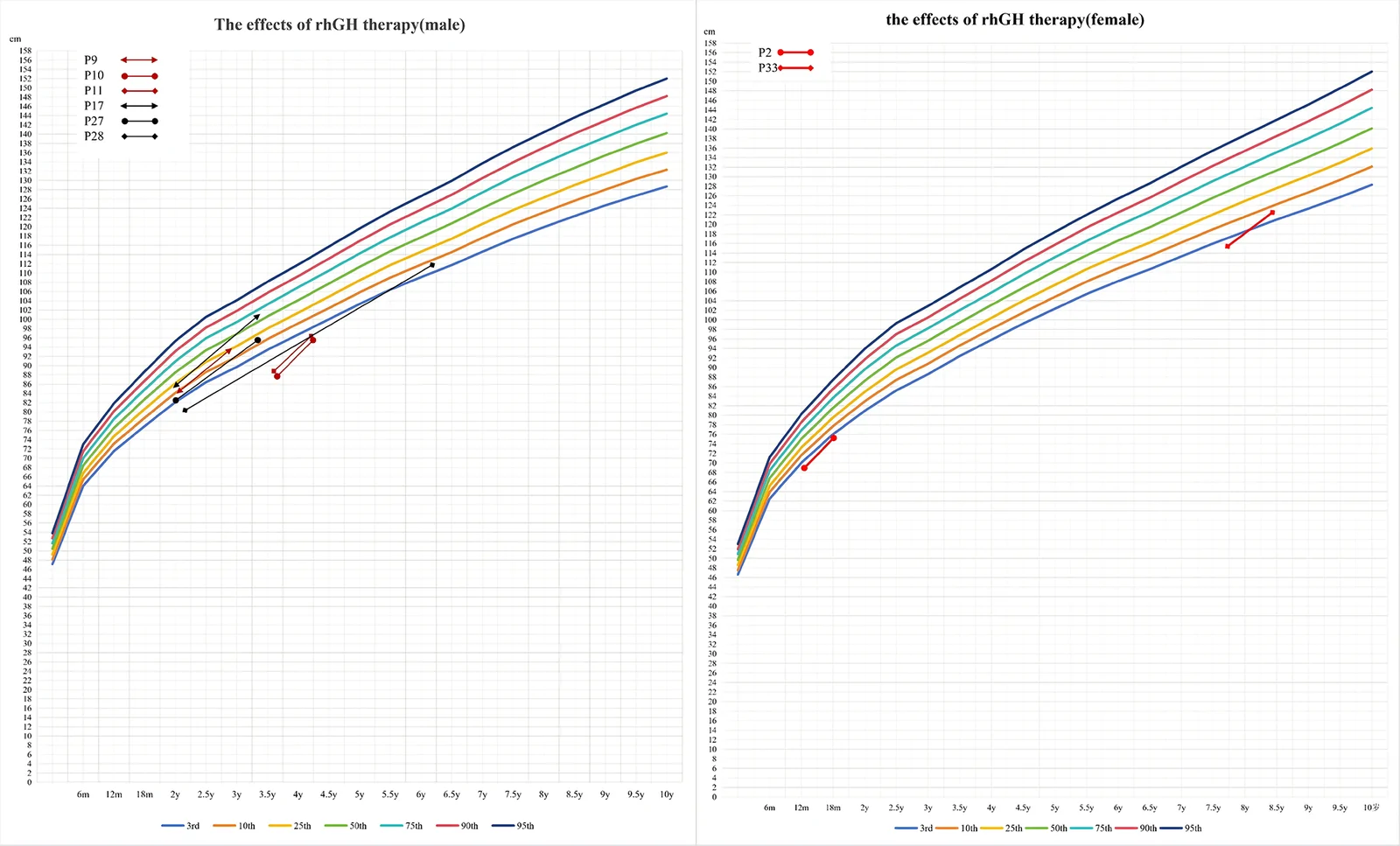
Effects of recombinant human growth hormone therapy

In our patient cohort, eight patients with or without growth hormone deficiency were treated with rhGH, and their height improved. The treatment duration ranged from 6 months to 4 years (Fig. 3; Table 3).

**Table 3:** Comparison before and after application of recombinant human growth hormone

Patient ID (Gender) Follow-up items		P2(F)	P9(M)	P10(M)	P11(M)	P17(M)	P27(M)	P28(M)	P33(F)
pre-treatment	Age	1y1m	2y	3y8m	3y8m	1y11m	2y	2y2m	7y9m
	Height(cm)	68	84	88	89	85	83	80.5	115.8
		(-2.59SD)	(-1.27SD)	(-3.33SD)	(-3.08SD)	(-0.88SD)	(-1.56SD)	(-2.60SD)	(-2.23SD)
	Weight(kg)	8.23	8.8	12	12.5	10.3	9.1	10	19.9
	IGF-1(ng/mL)	29.3	101.5	107.2	97.4	92.5	88.3	77.6	166.2
	The peak GH responses to levodopa and insulin(ng/mL)	NA	NA	NA	NA	NA	NA	4.07/6.79	25.4/22.1
	Bone age	0.5y	1.5y	2.5y	2.5y	NA	NA	1.5y	6y
	Developmental evaluation	DQ(89)	DQ(84)	−	−	DQ(85)	DQ(86)	DQ(61)	IQ(88.9)
post-treatment	Age	1y7m	2y11m	4y3m	4y3m	3y5m	3y4m	6y2m	8y4m
	Height(cm)	76	94	94.5	96	101	97.7	111.2	122
		(-2.00SD)	(-0.73SD)	(-2.56SD)	(-2.20SD)	(+ 0.26SD)	(-0.34SD)	(-1.64SD)	(-1.56SD)
	Weight(kg)	9	11.5	13.5	14.5	15	10.5	20.5	22.5
	IGF-1(ng/mL)	100.2	112.7	137.1	145.3	135.9	109.8	201.9	306.4
	Bone age	1y	2.5y	3y	3y	NA	NA	5.5y	7y
	Developmental evaluation	−	DQ(86)	−	−	DQ(97)	DQ(89)	DQ(78)	−

NA, Not Applicable; −,Unremarkable on initial assessment

## Discussion

In this study, we recruited 33 new Chinese patients with KBGS and conducted a retrospective analysis of data of 32 Chinese patients and 519 non-Chinese patients. To the best of our knowledge, this is the largest cohort of Chinese patients with KBGS reported to date, as well as the largest cohort of non-Chinese patients involved in a retrospective analysis. Previous studies have indicated insufficient correlation between racial groups. Therefore, in this retrospective study, we aimed to explore the relationship between different ethnic groups and the genotype–phenotype of the specific variation c.1903_1907del.

A retrospective analysis revealed that KBGS primarily results in three conditions: characteristic facial features, skeletal dysplasia, and abnormal nervous system development, which included developmental delay, macrodontia, brachydactyly and/or clinodactyly, large and/or prominent ears, abnormal cerebral MRI findings, visual complications, and short stature (≤ p3) [2, 4, 7–60]. The findings are consistent with those of previous studies. However, our study showed that the three conditions differ slightly between Chinese patients and patients of other countries. In Chinese patients, large and/or prominent ears were more common, whereas low hairline, triangular face, low-set ear, characteristic eyebrows (synophrys, bushy and/or thick eyebrows), anteverted nares, and long philtrum were less common. Therefore, we recommend that large and/or prominent ears be included in the characteristic features of Chinese patients among the major diagnostic criteria; low hairline, triangular face, characteristic eyebrows, anteverted nares, and long philtrum might not be suitable as the primary diagnostic feature in these patients. With respect to the skeletal system, Chinese patients were more likely to have large or delayed closure of the anterior fontanel and delayed bone age, a low incidence of macrodontia, short neck webbed neck, and spinal anomaly. The incidence of spinal abnormalities was low in all patients, possibly because some patients did not undergo spinal examination, and whether spinal abnormalities are a secondary diagnostic criterion requires deliberation. Regarding the nervous system, the observed lower prevalence of ID, epilepsy, and developmental delay in Chinese patients suggests a potentially milder degree of nervous system involvement in this cohort. However, it is important to consider that this finding could be influenced by the younger average age at assessment in our study group. The incidence of recurrent otitis media in Chinese patients was lower than that in patients of other countries, and therefore, recurrent otitis media might not be suitable as a diagnostic criterion for Chinese patients. Most Chinese patients have *de novo* variations, which may result in somatic mosaicism with *ANKRD11* variations [5]. Therefore, considering first-degree relatives with KBGS to be a primary diagnostic criterion seems inappropriate. Summarising the characteristics of Chinese patients, we found that delayed bone age, large and/or prominent ears, macrodontia, developmental delay, and brachydactyly and/or clinodactyly may be their primary diagnostic bases. We suggest that other characteristic facial features, abnormal cerebral MRI findings, short stature, large or delayed closure of anterior fontanelle, feeding difficulties, attention-deficit hyperactivity disorder (ADHD) symptoms, abnormal EEG, congenital heart defects, visual complications, and hearing loss should be considered as secondary diagnostic criteria for Chinese patients. The clinical diagnosis of KBGS is particularly challenging in the first few years of life because of the usually mild facial phenotype and the lack of major clinical signs [9]; however, large and/or prominent ears and large or delayed closure of anterior fontanelle are present early in life and are critical for the early diagnosis of KBGS. The impression from previous data is that some facial features can evolve in childhood and become more pronounced over time [9]. The atypical facial features of our patients may be related to their younger age, and whether this applies to older Chinese patients requires follow-up.

Notably, different races and genotypes have varying effects on diseases. Non-Chinese patients carrying c.1903_1907del in previous studies [9, 21–23, 25, 27, 31] were less likely to develop EEG abnormalities and less likely to develop hearing loss than Chinese patients carrying the same gene [7]. Studies have reported contradicting findings regarding the effect of c.1903_1907del on hearing [9], ranging from mild to severe, with some patients experiencing progressive severity with age. Therefore, the incidence of hearing loss in Chinese patients is likely to be underestimated and requires continuous follow-up. In contrast, non-Chinese patients carrying c.1903_1907del were more likely to exhibit a large anterior fontanelle or delayed closure but less likely to display thick and/or thick brows, thin upper lip, developmental delay, congenital heart defects, and vision complications. A study suggested a lower frequency of ID/ADHD/ASD among non-Chinese patients carrying the c.1903_1907del variant [10]; however, there were no differences after expanding the cohort. Among Chinese patients with the c.1903_1907del variant, a higher likelihood of epilepsy and a lower likelihood of delayed bone age were observed. A study suggested no association between epilepsy and genotype [57]; our findings may have been affected by the small cohort size. The previous study reported the effects of the c.1903_1907del variant on the skeletal system, nervous system, and facial features of different populations, suggesting that the c.1903_1907del variant can cause unique phenotypes, which may be influenced by race and epigenetics.

The incidence of IUGR, polyhydramnios, abnormal renal and brain morphology, abnormal cardiovascular system morphology, preterm birth, and higher NT were higher in Chinese and non-Chinese than in the general population (8.47%–11.1% (*P* ≤ 10) [61–63], 0.2%–2% [64], 0.9% [65], 0.5% [65], 9.26%–10.23% [62, 66–68], and 2.7% [69], respectively). Therefore, we speculate that patients with KBGS are more susceptible to these prenatal challenges. When these features are identified, especially as a constellation of findings, it may be appropriate to consider KBGS in the differential diagnosis. In such scenarios, and particularly when there is a relevant family history, referral for genetic counseling to discuss the option of testing for ANKRD11 variants and 16q24.3 microdeletions is warranted.

We identified some rare phenotypes, including hypoplasia of the phalanges of the fourth toe, persistent hyperplasia of the primary vitreous, familial exudative vitreoretinopathy, Mongolian spot, leucoplakia, severe eczema, keratosis follicularis, and hepatosplenomegaly. It is noteworthy that whether these represent rare features of the KBGS spectrum or coincidental findings remains to be elucidated in larger studies.Epileptic phenotypes in KBGS encompass systemic and focal seizures, with individual patients exhibiting different epileptic phenotypes [70]. Tonic-clonic seizures are the predominant seizure type described in the literature and observed in our case; however, no characteristic seizure phenotype associated with KBGS has been identified [70]. Irrespective of the new patients included in our study or the patients involved in previous studies, some of them have abnormal EEG finding but no seizures [51]. Therefore, consideration of an EEG at diagnosis, guided by clinical judgment and in consultation with a paediatric neurologist, may be beneficial for some patients. Our study and previous studies show that the seizure forms of the same genotype varied. Moreover, significant differences exist in the incidence rate of epilepsy with the same genotype among different ethnic groups, and variations are also observed in the incidence rate of epilepsy with different genotypes among Chinese patients [7, 9, 10, 21–23]. Most of our patients experienced spontaneous remission of epilepsy or responded well to medication, and this is consistent with previous literature reports; while various anti-seizure medications were used effectively, no single medication was superior across the cohort, reflecting the heterogeneous nature of seizures in KBGS [70]. Infantile spasms have been reported, drug resistance to antiepileptic therapy has been observed, and the evolution of epilepsy phenotypes is diverse [51]. The symptoms in our patients were relieved after treatment with sodium valproate; however, because of the young age of the patients and the short follow-up time, epilepsy control should be considered. LGS has rarely been reported in patients with KBGS, and treatment and follow-up are limited [70]. The therapeutic outcomes in our patients were poor; a long-term follow-up will help determine whether they achieve controlled epilepsy status.

KBGS is associated with IUGR and postnatal growth retardation, and gradual deviation from the genetic curve is among its primary manifestations [2]. In 2015, Reynaert et al. [71] reported the response of patients with KBGS to rhGH treatment for the first time and proposed that treatment with exogenous growth hormone should be effective for short stature in children with KBG syndrome regardless of GH deficiency. Furthermore, preliminary studies [6, 12, 13] have shown that rhGH treatment can effectively improve the height of short children with KBGS, consistent with the findings of previous studies. ANKRD11 can affect cartilage differentiation, and growth hormones may promote cartilage differentiation through a mechanism that improves height [4]. The efficacy and safety of rhGH are consistent with those reported previously [13]; However, given the limited cohort size and follow-up duration, it remains unknown whether these height gains will be maintained. The long-term efficacy and safety require validation through future studies with extended observation.

This study has few limitations. KBGS involves multiple systems, such as nervous, skeletal, and facial systems, which can easily be overlooked in the evaluation. Although we conducted a systematic evaluation of most patients, our patients are relatively young and the follow-up period was short. Some phenotypes may change with age, and currently, the phenotype data for different age groups are less comprehensive. In addition, some patient evaluations reported previously were limited, and we assumed them to be none during the statistical analysis, which may affect the statistical results. However, we established the largest cohort of Chinese patients, proposed the differences among different races for the first time, and attempted to establish diagnostic criteria for Chinese patients, which are of great significance. We will continue to follow up the new patients through face-to-face consultations or video conferences, regularly improve examinations, and attempt to obtain data from various age groups, providing more basis for the establishment of standards for Chinese patients.

This study, by characterizing the largest cohort of Chinese KBGS patients to date, addresses a critical gap arising from the fact that current diagnostic criteria are primarily based on Caucasian populations. We demonstrate that while the core features of KBGS are conserved, key diagnostic facial features (e.g., triangular face, synophrys) are less common in Chinese patients, whereas large/prominent ears are a more prevalent hallmark. These ethnic-specific phenotypic differences are crucial for clinicians to recognize, as they can significantly improve diagnostic accuracy and prevent under-diagnosis in Chinese populations. Furthermore, our data contribute to the growing evidence on the prenatal phenotype of KBGS and the medium-term outcomes of GH therapy. Future research should focus on validating ethnic-specific diagnostic guidelines and establishing the long-term efficacy and safety of treatments across diverse populations to optimize global patient care.

## Supplementary Information

Below is the link to the electronic supplementary material.

Supplementary Material 1: Detailed information of the questionnaires of 33 patients

Supplementary Material 2: Comparison of perinatal problems between the Chinese and non-Chinese patients

Supplementary Material 3: Chinese Patient Gene

## Data Availability

The datasets used and/or analysed during the current study are available from the corresponding author on reasonable request.

## Abbreviations

ACMG: American College of Medical Genetics and Genomics
ADHD: Attention-deficit hyperactivity disorder
ANKRD11: Ankyrin repeat domain 11
ASD: Autism spectrum disorder
EEG: Electroencephalogram
ID: Intellectual disability
IUGR: Intrauterine growth retardation
KBGS: KBG syndrome
LGS: Lennox-Gastaut syndrome
MRI: Magnetic resonance imaging
NT: Nuchal translucency
PLSVC: Persistent left superior vena cava
rhGH: Recombinant human growth hormone
VSD: Ventricular septal defect
WES: Whole exome sequencing

## References

[1] Herrmann J, Pallister PD, Tiddy W, Opitz JM. The KBG syndrome-a syndrome of short stature, characteristic facies, mental retardation, macrodontia and skeletal anomalies. Birth Defects Orig Artic Ser. 1975;11:7–18. 1218237

[2] Sirmaci A, Spiliopoulos M, Brancati F, Powell E, Duman D, Abrams A, et al. Mutations in ANKRD11 cause KBG syndrome, characterized by intellectual disability, skeletal malformations, and macrodontia. Am J Hum Genet. 2011;89:289–94. 10.1016/j.ajhg.2011.06.007. PMC3155157 21782149

[3] Gallagher D, Voronova A, Zander MA, Cancino GI, Bramall A, Krause MP, et al. Ankrd11 is a chromatin regulator involved in autism that is essential for neural development. Dev Cell. 2015;32:31–42. 10.1016/j.devcel.2014.11.031. 25556659

[4] Zhang T, Yang Y, Yin X, Wang X, Ni J, Dong Z, et al. Two loss-of-function ANKRD11 variants in Chinese patients with short stature and a possible molecular pathway. Am J Med Genet A. 2021;185:710–8. 10.1002/ajmg.a.62024. PMC7898801 33354850

[5] Crippa M, Rusconi D, Castronovo C, Bestetti I, Russo S, Cereda A, et al. Familial intragenic duplication of ANKRD11 underlying three patients of KBG syndrome. Mol Cytogenet. 2015;8:20. 10.1186/s13039-015-0126-7. PMC4383199 25838844

[6] Morel Swols D, Foster J, Tekin M. KBG syndrome. Orphanet J Rare Dis. 2017;12:183. 10.1186/s13023-017-0736-8. PMC5735576 29258554

[7] Gao F, Zhao X, Cao B, Fan X, Li X, Li L, et al. Genetic and phenotypic spectrum of KBG syndrome: A report of 13 new Chinese cases and a review of the literature. J Pers Med. 2022;12:407. 10.3390/jpm12030407. PMC8948816 35330407

[8] Ho S, Luk HM, Lo IFM. KBG syndrome in a Chinese population: A case series. Am J Med Genet A. 2022;188:1693–9. 10.1002/ajmg.a.62688. 35174959

[9] Low K, Ashraf T, Canham N, Clayton-Smith J, Deshpande C, Donaldson A, et al. Clinical and genetic aspects of KBG syndrome. Am J Med Genet A. 2016;170:2835–46. 10.1002/ajmg.a.37842. PMC5435101 27667800

[10] Martinez-Cayuelas E, Blanco-Kelly F, Lopez-Grondona F, Swafiri ST, Lopez-Rodriguez R, Losada-Del Pozo R, et al. Clinical description, molecular delineation and genotype-phenotype correlation in 340 patients with KBG syndrome: Addition of 67 new patients. J Med Genet. 2023;60:644–54. 10.1136/jmg-2022-108632. 36446582

[11] Xu M, Zhou H, Yong J, Cong P, Li C, Yu Y, et al. A Chinese patient with KBG syndrome and a 9q31.2-33.1 microdeletion. Eur J Med Genet. 2013;56:245–50. 10.1016/j.ejmg.2013.01.007. 23369839

[12] Kang Y, He D, Li Y, Zhang Y, Shao Q, Zhang M, et al. A heterozygous point mutation of the ANKRD11 (c.2579C > T) in a Chinese patient with idiopathic short stature. Mol Genet Genomic Med. 2019;7:e988. 10.1002/mgg3.988. PMC6900381 31566922

[13] Ge XY, Ge L, Hu WW, Li XL, Hu YY. Growth hormone therapy for children with KBG syndrome: A case report and review of literature. World J Clin Cases. 2020;8:1172–9. 10.12998/wjcc.v8.i6.1172. PMC7103963 32258089

[14] Chen J, Xia Z, Zhou Y, Ma X, Wang X, Guo Q. A de novo frameshift variant of ANKRD11 (c.1366_1367dup) in a Chinese patient with KBG syndrome. BMC Med Genomics. 2021;14:68. 10.1186/s12920-021-00920-3. PMC7927266 33653342

[15] Li Q, Sun C, Yang L, Lu W, Luo F. Comprehensive analysis of clinical spectrum and genotype associations in Chinese and literature reported KBG syndrome. Transl Pediatr. 2021;10:834–42. 10.21037/tp-20-385. PMC8107870 34012832

[16] Deng T, Liu Q, Xie J, Li X, Yao B. A case of prenatal diagnosis of 16q24.3 microdeletion KBG syndrome and review of the literature. Clin Case Rep. 2022;10:e5958. 10.1002/ccr3.5958. PMC9207229 35765297

[17] Sacharow S, Li D, Fan YS, Tekin M. Familial 16q24.3 microdeletion involving ANKRD11 causes a KBG-like syndrome. Am J Med Genet A. 2012;158A:547–52. 10.1002/ajmg.a.34436. 22307766

[18] Khalifa M, Stein J, Grau L, Nelson V, Meck J, Aradhya S, et al. Partial deletion of ANKRD11 results in the KBG phenotype distinct from the 16q24.3 microdeletion syndrome. Am J Med Genet A. 2013;161A:835–40. 10.1002/ajmg.a.35739. 23494856

[19] Miyatake S, Murakami A, Okamoto N, Sakamoto M, Miyake N, Saitsu H, et al. A de novo deletion at 16q24.3 involving ANKRD 11 in a Japanese patient with KBG syndrome. Am J Med Genet A. 2013;161:1073–7. 10.1002/ajmg.a.35661. 23463723

[20] Lim JH, Seo EJ, Kim YM, Cho HJ, Lee JO, Cheon CK, et al. A de novo microdeletion of ANKRD11 gene in a Korean patient with KBG syndrome. Ann Lab Med. 2014;34:390–4. 10.3343/alm.2014.34.5.390. PMC4151010 25187894

[21] Ockeloen CW, Willemsen MH, de Munnik S, van Bon BWM, de Leeuw N, Verrips A, et al. Further delineation of the KBG syndrome phenotype caused by ANKRD11 aberrations. Eur J Hum Genet. 2015;23:1176–85. 10.1038/ejhg.2014.253. PMC4538199 25424714

[22] Goldenberg A, Riccardi F, Tessier A, Pfundt R, Busa T, Cacciagli P, et al. Clinical and molecular findings in 39 patients with KBG syndrome caused by deletion or mutation of ANKRD11. Am J Med Genet A. 2016;170:2847–59. 10.1002/ajmg.a.37878. 27605097

[23] Murray N, Burgess B, Hay R, Colley A, Rajagopalan S, McGaughran J, et al. KBG syndrome: An Australian experience. Am J Med Genet A. 2017;173:1866–77. 10.1002/ajmg.a.38121. 28449295

[24] Scarano E, Tassone M, Graziano C, Gibertoni D, Tamburrino F, Perri A, et al. Novel mutations and unreported clinical features in KBG syndrome. Mol Syndromol. 2019;10:130–8. 10.1159/000496172. PMC6528090 31191201

[25] Kutkowska-Kaźmierczak A, Boczar M, Kalka E, Castañeda J, Klapecki J, Pietrzyk A, et al. Wide fontanels, delayed speech development and hoarse voice as useful signs in the diagnosis of KBG syndrome: A clinical description of 23 cases with pathogenic variants involving the ANKRD11 gene or submicroscopic chromosomal rearrangements of 16q24.3. Genes (Basel). 2021;12:1257. 10.3390/genes12081257. PMC8394041 34440431

[26] Kim HJ, Cho E, Park JB, Im WY, Kim HJ. A Korean family with KBG syndrome identified by ANKRD11 mutation, and phenotypic comparison of ANKRD11 mutation and 16q24.3 microdeletion. Eur J Med Genet. 2015;58:86–94. 10.1016/j.ejmg.2014.11.003. 25464108

[27] Walz K, Cohen D, Neilsen PM, Foster J, Brancati F, Demir K, et al. Characterization of ANKRD11 mutations in humans and mice related to KBG syndrome. Hum Genet. 2015;134:181–90. 10.1007/s00439-014-1509-2. 25413698

[28] Kleyner R, Malcolmson J, Tegay D, Ward K, Maughan A, Maughan G, et al. KBG syndrome involving a single-nucleotide duplication in ANKRD11. Cold Spring Harb Mol Case Stud. 2016;2:a001131. 10.1101/mcs.a001131. PMC5111005 27900361

[29] Parenti I, Gervasini C, Pozojevic J, Graul-Neumann L, Azzollini J, Braunholz D, et al. Broadening of cohesinopathies: Exome sequencing identifies mutations in ANKRD11 in two patients with Cornelia de Lange-overlapping phenotype. Clin Genet. 2016;89:74–81. 10.1111/cge.12564. 25652421

[30] Low KJ, Hills A, Williams M, Duff-Farrier C, McKee S, Smithson SF. A splice-site variant in ANKRD11 associated with classical KBG syndrome. Am J Med Genet A. 2017;173:2844–6. 10.1002/ajmg.a.38397. 28815928

[31] Miyatake S, Okamoto N, Stark Z, Nabetani M, Tsurusaki Y, Nakashima M, et al. ANKRD11 variants cause variable clinical features associated with KBG syndrome and Coffin-Siris-like syndrome. J Hum Genet. 2017;62:741–6. 10.1038/jhg.2017.24. PMC5537415 28250421

[32] Novara F, Rinaldi B, Sisodiya SM, Coppola A, Giglio S, Stanzial F, et al. Haploinsufficiency for ANKRD11-flanking genes makes the difference between KBG and 16q24.3 microdeletion syndromes: 12 new cases. Eur J Hum Genet. 2017;25:694–701. 10.1038/ejhg.2017.49. PMC5533198 28422132

[33] Srivastava P, Gambhir PS, Phadke SR. KBG syndrome: 16q24.3 microdeletion in an Indian patient. Clin Dysmorphol. 2017;26:161–6. 10.1097/MCD.0000000000000168. 28099180

[34] Crippa M, Bestetti I, Maitz S, Weiss K, Spano A, Masciadri M, et al. SETD5 gene haploinsufficiency in three patients with suspected KBG syndrome. Front Neurol. 2020;11:631. 10.3389/fneur.2020.00631. PMC7393934 32793091

[35] Palumbo O, Palumbo P, Di Muro E, Cinque L, Petracca A, Carella M, et al. A private 16q24.2q24.3 Microduplication in a Boy with Intellectual Disability, Speech Delay and Mild Dysmorphic Features. Genes (Basel). 2020;11:707. 10.3390/genes11060707. PMC7349372 32604767

[36] Bianchi PM, Bianchi A, Digilio MC, Tucci FM, Sitzia E, De Vincentiis GC. Audiological findings in a de novo mutation of ANKRD11 gene in KBG syndrome: Report of a case and review of the literature. Int J Pediatr Otorhinolaryngol. 2017;103:109–12. 10.1016/j.ijporl.2017.10.017. 29224748

[37] Reuter MS, Chaturvedi RR, Liston E, Manshaei R, Aul RB, Bowdin S, et al. The Cardiac Genome Clinic: Implementing genome sequencing in pediatric heart disease. Genet Med. 2020;22:1015–24. 10.1038/s41436-020-0757-x. PMC7272322 32037394

[38] van Dongen LCM, Wingbermühle E, van der Veld WM, Vermeulen K, Bos-Roubos AG, Ockeloen CW, et al. Exploring the behavioral and cognitive phenotype of KBG syndrome. Genes Brain Behav. 2019;18:e12553. 10.1111/gbb.12553. PMC6850621 30786142

[39] Sayed ISM, Abdel-Hamid MS, Abdel-Salam GMH. KBG syndrome in two patients from Egypt. Am J Med Genet A. 2020;182:1309–12. 10.1002/ajmg.a.61552. 32222090

[40] Behnert A, Auber B, Steinemann D, Frühwald MC, Huisinga C, Hussein K, et al. KBG syndrome patient due to 16q24.3 microdeletion presenting with a paratesticular rhabdoid tumor: Coincidence or cancer predisposition? Am J Med Genet A. 2018;176:1449–54. 10.1002/ajmg.a.38724. 29696793

[41] Libianto R, Wu KH, Devery S, Eisman JA, Center JR. KBG syndrome presenting with brachydactyly type E. Bone. 2019;123:18–22. 10.1016/j.bone.2019.03.012. 30877071

[42] Gnazzo M, Lepri FR, Dentici ML, Capolino R, Pisaneschi E, Agolini E, et al. KBG syndrome: Common and uncommon clinical features based on 31 new patients. Am J Med Genet A. 2020;182:1073–83. 10.1002/ajmg.a.61524. 32124548

[43] Alves RM, Uva P, Veiga MF, Oppo M, Zschaber FCR, Porcu G, et al. Novel ANKRD11 gene mutation in an individual with a mild phenotype of KBG syndrome associated to a GEFS+ phenotypic spectrum: A case report. BMC Med Genet. 2019;20:16. 10.1186/s12881-019-0745-7. PMC6332862 30642272

[44] De Bernardi ML, Ivanovski I, Caraffi SG, Maini I, Street ME, Bayat A, et al. Prominent and elongated coccyx, a new manifestation of KBG syndrome associated with novel mutation in ANKRD11. Am J Med Genet A. 2018;176:1991–5. 10.1002/ajmg.a.40386. 30088855

[45] Kim SJ, Yang A, Park JS, Kwon DG, Lee JS, Kwon YS, et al. Two novel mutations of ANKRD11 gene and wide clinical spectrum in KBG syndrome: Case reports and literature review. Front Genet. 2020;11:579805. 10.3389/fgene.2020.579805. PMC7687677 33262785

[46] Willemsen MH, Fernandez BA, Bacino CA, Gerkes E, de Brouwer APM, Pfundt R, et al. Identification of ANKRD11 and ZNF778 as candidate genes for autism and variable cognitive impairment in the novel 16q24.3 microdeletion syndrome. Eur J Hum Genet. 2010;18:429–35. 10.1038/ejhg.2009.192. PMC2987261 19920853

[47] Youngs EL, Hellings JA, Butler MG. ANKRD11 gene deletion in a 17-year-old male. Clin Dysmorphol. 2011;20:170–1. 10.1097/MCD.0b013e328346f6ae. PMC5283804 21527850

[48] Isrie M, Hendriks Y, Gielissen N, Sistermans EA, Willemsen MH, Peeters H, et al. Haploinsufficiency of ANKRD11 causes mild cognitive impairment, short stature and minor dysmorphisms. Eur J Hum Genet. 2012;20:131–3. 10.1038/ejhg.2011.105. PMC3260937 21654729

[49] Cucco F, Sarogni P, Rossato S, Alpa M, Patimo A, Latorre A, et al. Pathogenic variants in EP300 and ANKRD11 in patients with phenotypes overlapping Cornelia de Lange syndrome. Am J Med Genet A. 2020;182:1690–6. 10.1002/ajmg.a.61611. 32476269

[50] Mattei D, Cavarzere P, Gaudino R, Antoniazzi F, Piacentini G. Dysmorphic features and adult short stature: Possible clinical markers of KBG syndrome. Ital J Pediatr. 2021;47:15. 10.1186/s13052-021-00961-5. PMC7830821 33494799

[51] Nardello R, Mangano GD, Antona V, Fontana A, Striano P, Giorgio E, et al. Electroclinical features and outcome of ANKRD11-related KBG syndrome: A novel report and literature review. Seizure. 2021;85:151–4. 10.1016/j.seizure.2020.12.017. 33476899

[52] Parenti I, Mallozzi MB, Hüning I, Gervasini C, Kuechler A, Agolini E, et al. ANKRD11 variants: KBG syndrome and beyond. Clin Genet. 2021;100:187–200. 10.1111/cge.13977. 33955014

[53] Borja N, Zafeer MF, Rodriguez JA, Morel Swols D, Thorson W, Bademci G, et al. Deletion of first noncoding exon in ANKRD11 leads to KBG syndrome. Am J Med Genet A. 2023;191:1044–9. 10.1002/ajmg.a.63119. 36628575

[54] Choi Y, Choi J, Do H, Hwang S, Seo GH, Choi IH, et al. KBG syndrome: Clinical features and molecular findings in seven unrelated Korean families with a review of the literature. Mol Genet Genomic Med. 2023;11:e2127. 10.1002/mgg3.2127. PMC10094073 36564961

[55] Guo L, Park J, Yi E, Marchi E, Hsieh TC, Kibalnyk Y, et al. KBG syndrome: Videoconferencing and use of artificial intelligence driven facial phenotyping in 25 new patients. Eur J Hum Genet. 2022;30:1244–54. 10.1038/s41431-022-01171-1. PMC9626563 35970914

[56] Loberti L, Bruno LP, Granata S, Doddato G, Resciniti S, Fava F, et al. Natural history of KBG syndrome in a large European cohort. Hum Mol Genet. 2022;31:4131–42. 10.1093/hmg/ddac167. PMC9759332 35861666

[57] Buijsse N, Jansen FE, Ockeloen CW, van Kempen MJA, Zeidler S, Willemsen MH, et al. Epilepsy is an important feature of KBG syndrome associated with poorer developmental outcome. Epilepsia Open. 2023;8:1300–13. 10.1002/epi4.12799. PMC10690702 37501353

[58] De Boer E, Ockeloen CW, Kampen RA, Hampstead JE, Dingemans AJM, Rots D, et al. Missense variants in ANKRD11 cause KBG syndrome by impairment of stability or transcriptional activity of the encoded protein. Genet Med. 2022;24:2051–64. 10.1016/j.gim.2022.06.007. 35833929

[59] Peluso F, Caraffi SG, Contrò G, Valeri L, Napoli M, Carboni G, et al. Deep phenotyping of the neuroimaging and skeletal features in KBG syndrome: A study of 53 patients and review of the literature. J Med Genet. 2023;60:1224–34. 10.1136/jmg-2023-109141. PMC10715526 37586838

[60] Amllal N, Elalaoui SC, Zerkaoui M, Chiguer A, Afif L, Izgua AT, et al. Identification of two novel ANKRD11 mutations: Highlighting incomplete penetrance in KBG syndrome. Ann Lab Med. 2024;44:110–7. 10.3343/alm.2024.44.1.110. PMC10485853 37665295

[61] Jensen EA, Foglia EE, Dysart KC, Simmons RA, Aghai ZH, Cook A, et al. Adverse effects of small for gestational age differ by gestational week among very preterm infants. Arch Dis Child Fetal Neonatal Ed. 2019;104:F192–8. 10.1136/archdischild-2017-314171. PMC6335180 29730594

[62] Harrison W, Goodman D. Epidemiologic trends in neonatal intensive care, 2007–2012. JAMA Pediatr. 2015;169:855–62. 10.1001/jamapediatrics.2015.1305. 26214387

[63] Pagani G, D’Antonio F, Khalil A, Akolekar R, Papageorghiou A, Bhide A, et al. Association between reduced fetal movements at term and abnormal uterine artery Doppler indices. Ultrasound Obstet Gynecol. 2014;43:548–52. 10.1002/uog.13220. 24123633

[64] Matěcha J, Nováčková M. Idiopathic polyhydramnios. Ceska Gynekol. 2020;85:417–21. 33711902

[65] Drukker L, Cavallaro A, Salim I, Ioannou C, Impey L, Papageorghiou AT. How often do we incidentally find a fetal abnormality at the routine third-trimester growth scan? A population-based study. Am J Obstet Gynecol. 2020;223:e9191–91913. 10.1016/j.ajog.2020.05.052. 32504567

[66] Martin JA, Hamilton BE, Osterman MJK, Driscoll AK. Births: Final data for 2019. Natl Vital Stat Rep. 2021;70:1–51. 33814033

[67] Martin JA, Hamilton BE, Osterman MJ. Births in the United States, 2021. NCHS Data Brief. 2022;(442):1–8. 36043891

[68] Purisch SE, Gyamfi-Bannerman C. Epidemiology of preterm birth. Semin Perinatol. 2017;41:387–91. 10.1053/j.semperi.2017.07.009. 28865982

[69] Bellai-Dussault K, Dougan SD, Fell DB, Little J, Meng L, Okun N, et al. Ultrasonographic fetal nuchal translucency measurements and cytogenetic outcomes. JAMA Netw Open. 2024;7:e243689. 10.1001/jamanetworkopen.2024.3689. PMC10966411 38530313

[70] Auconi M, Serino D, Digilio MC, Gnazzo M, Conti M, Vigevano F, et al. Epilepsy in KBG syndrome. Dev Med Child Neurol. 2023;65:712–20. 10.1111/dmcn.15428. 36196002

[71] Reynaert N, Ockeloen CW, Sävendahl L, Beckers D, Devriendt K, Kleefstra T, et al. Short stature in KBG syndrome: First responses to growth hormone treatment. Horm Res Paediatr. 2015;83:361–4. 10.1159/000380908. 25833229

